# An Integrative Bioinformatics Analysis of the Potential Mechanisms Involved in Propofol Affecting Hippocampal Neuronal Cells

**DOI:** 10.1155/2022/4911773

**Published:** 2022-04-26

**Authors:** Zhao Zhuang, Dajiang Li, Mengmeng Jiang, Ye Wang, Qianqian Cao, Shenfeng Li, Ruixue Luan, Lina Sun, Shoushi Wang

**Affiliations:** ^1^Academy of Anesthesiology, Weifang Medical University, Weifang, China; ^2^Qingdao Central Hospital, Central Hospital Affiliated to Qingdao University, Qingdao, China; ^3^Academy of Anesthesiology, Qingdao University, Qingdao, China

## Abstract

The aim of this study is to probe the possible molecular mechanisms underlying the effects of propofol on HT22 cells. HT22 cells treated with different concentrations were sequenced, and then the results of the sequencing were analyzed for dynamic trends. Expression pattern clustering analysis was performed to demonstrate the expression of genes in the significant trend modules in each group of samples. We first chose the genes related to the trend module for WGCNA analysis, then constructed the PPI network of module genes related to propofol treatment group, and screened the key genes. Finally, GSEA analysis was performed on the key genes. Overall, 2,506 genes showed a decreasing trend with increasing propofol concentration, and 1,871 genes showed an increasing trend with increasing propofol concentration. WGCNA analysis showed that among them, turquoise panel genes were negatively correlated with propofol treatment, and genes with Cor R >0.9 in the turquoise panel were selected for PPI network construction. The MCC algorithm screened a total of five key genes (CD86, IL10RA, PTPRC, SPI1, and ITGAM). GSEA analysis showed that CD86, IL10RA, PTPRC, SPI1, and ITGAM are involved in the PRION_DISEASES pathway. Our study showed that propofol sedation can affect mRNA expression in the hippocampus, providing new ideas to identify treatment of nerve injury induced by propofol anesthesia.

## 1. Introduction

Propofol is one of the intravenous hypnotic medicines used to induce and maintain sedation and general anesthesia. It exerts its action through potentiation of the inhibitory neurotransmitter *γ*-aminobutyric acid at the GABA-A receptor [1, 2]. It is highly lipophilic and thus can rapidly cross the blood-brain barrier, leading to early onset of action. The characteristics of propofol are well known. It has fast metabolism and short recovery time, regardless of the depth or length of sedation time [3]. In addition to being used as an anesthetic, researchers also found that propofol is also related to many cancer-related pathophysiological processes and can play an important role in cancer by regulating the expression of a variety of downstream molecules, long-chain noncoding RNA, microRNA, and signal pathways [4]. Propofol upregulates miR-195, and then JAK/stat and NF-*κ*B pathway is inactivated to inhibit the proliferation, migration, and invasion of gastric carcinoma MKN45 cells [5].

Although propofol can play an excellent role in maintaining sedation and general anesthesia, studies have also focused on changes in regional cerebral blood flow or neuronal activity during propofol sedation. Only one study showed that as the depth of propofol sedation increased, the activity in the area corresponding to the stimulus applied during propofol sedation decreased. In addition, they found that propofol sedation impaired the function of basal ganglia circuits and thalamocortical connections [6]. It is understood that learning and memory occur mainly in the hippocampus, and propofol may cause hippocampal neurotoxicity [7, 8]. General anesthetics may induce developmental neurotoxicity, including subsequent long-term memory, acute extensive nerve cell death, and abnormal learning behavior [9]. Although many researchers have figured out that propofol induced neurotoxicity in the brains of developing animals, its exact mechanism of action remains largely unknown. Then, this manuscript, on the other hand, was conducted to observe the potential biological processes and signaling pathways that may play an important role during propofol sedation through the biology information analysis of genes with different expression patterns.

## 2. Methods

### 2.1. Cell Processing and Sequencing

The HT22 hippocampal cell line used in the present study was purchased from Shanghai Yaji Biotechnology Co. (Shanghai, China). The cells were fostered in DMEM containing 10% FBC, 100 *μ*/mL penicillin, 100 ng/mL streptomycin at 37°C, and 5% CO_2_. When the cell density reached 60–70%, the HT22 cells were exposed to propofol at concentrations of 25 *μ*g/ml, 50 *μ*g/ml, 75 *μ*g/ml, and 100 *μ*g/ml for 1 day. The experiment was repeated three times. The cDNA/DNA/Small RNA was sequenced on the Illumina sequencing platform by Genednovo Biotechnology (Guangzhou, China). The libraries were sequenced.

### 2.2. Effects of Treatment on CCK-8

**Figure 1:**
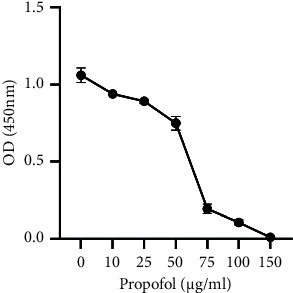
CCK8 shows the processing effect.

Cells were inoculated in 96-well plates at 1 × 10 3 cells per well. Cell Counting Kit-8 (CCK-8; Beyotime Biotechnology, Shanghai, China) assays were performed daily. Briefly, the cells were incubated in a 100 *μ*L medium containing 10 *μ*L of CCK-8 (0.5 mg/mL) reagent at 37°C for one to four hours. Absorbance was measured at 450 nm by an enzyme-labeled assay (Figure 1). The experiment was performed at least three times in three replicate wells.

### 2.3. Trend Analysis

The results obtained from sequencing were subjected to follow-up experiments. The expression of each gene was grouped according to different concentration points, and then a dynamic trend analysis was completed using OmicShare Tools. A *P* value <0.05 would show a significant difference.

### 2.4. Protein-Protein Interaction (PPI) Network Construction

All associations got from STRING were provided with a confidence score and imported into Cytoscape software. Modules of the PPI network were screened using the Molecular Complex Detection plugin in Cytoscape. A *P* value <0.05 would show a significant difference.

### 2.5. Gene Ontology (GO) Function

Gene ontology annotations and KEGG pathway enrichment analyses were performed using the Enrichr database GO terms consisting of the following three components: biological process (BP), cellular component (CC), and molecular function (MF).

### 2.6. WGCNA Analysis

Co-expression networks were constructed using the WGCNA data bank in R software. Paired Pearson correlations were first used to evaluate the weighted co-expression relationships between all subjects in the dataset in the adjacency matrix. The matrices were then converted to TOMs using the topological overlap matrix (TOM) similarity function, and the resulting TOMs were used to measure co-expression relationships between genes based on biologically meaningful genetic similarity.

### 2.7. GSEA Analysis

The genes were classified into high and low-expression parts, and then GSEA V3.0 software was used to analyze the enrichment results for the genes. A nominal *P* value of <0.05 and false discovery rate (FDR) of <25% were selected as cut-off criteria. We selected the top two ranked analysis results.

## 3. Results

### 3.1. Trend Analysis

**Figure 2:**
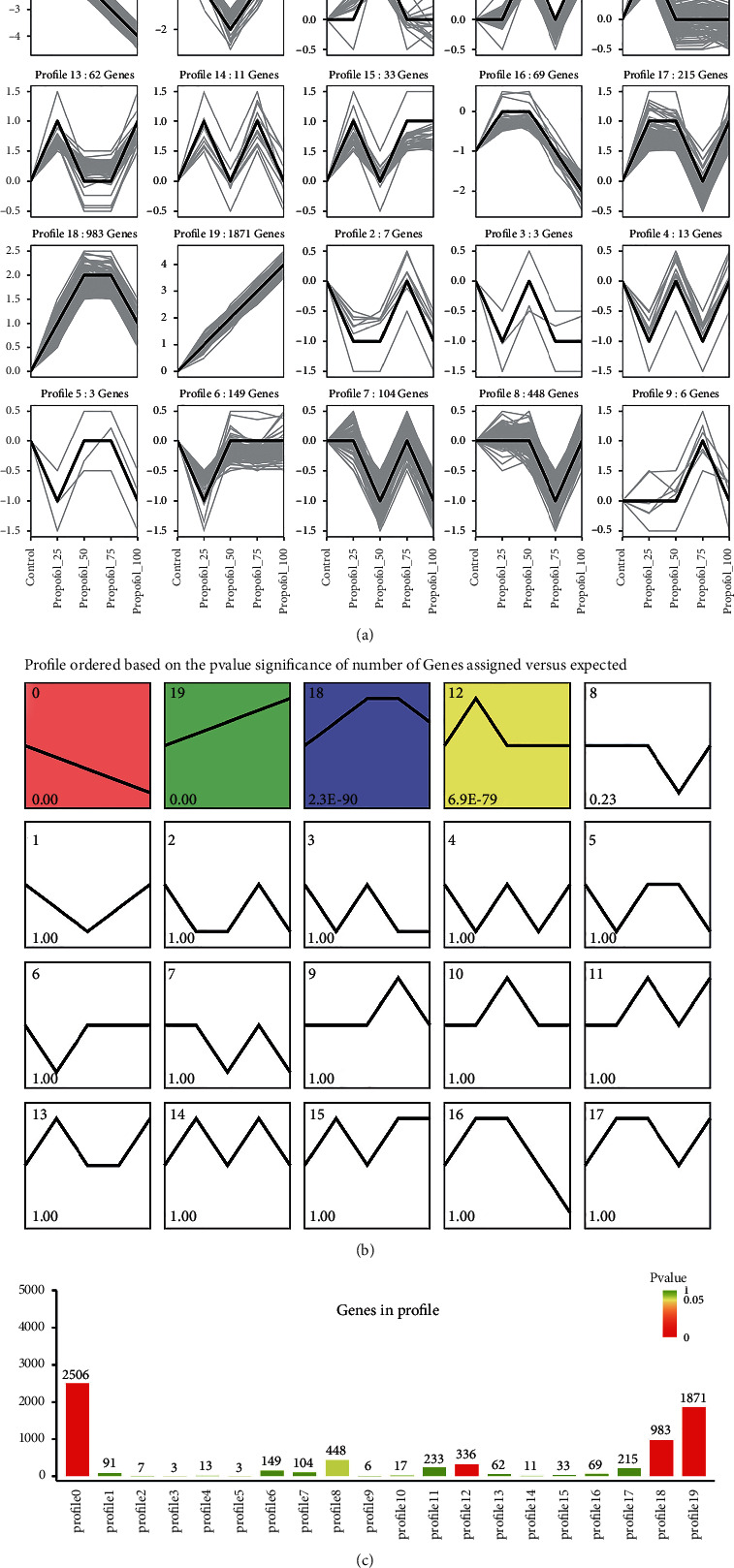
Trend analysis. (a) Number of genes in each module. (b) *P*-value of each module. (c) Bar graph showing the number of genes and *P*-value of each module.

We first subjected the expression data obtained from sequencing to trend analysis to observe the genes whose expression continued to increase or decrease with increasing propofol concentration. The results of the trend analysis showed that genes concentrated in module 0 decreased in expression with increasing propofol concentration, and genes concentrated in module 19 increased in expression with increasing propofol concentration; both panels were differentially significant. There were 2,506 genes in module 0 and 1,871 genes in module 19 (see Figure 2).

### 3.2. Heat Map Showing the Expression of Each Gene in Each Sample

**Figure 3:**
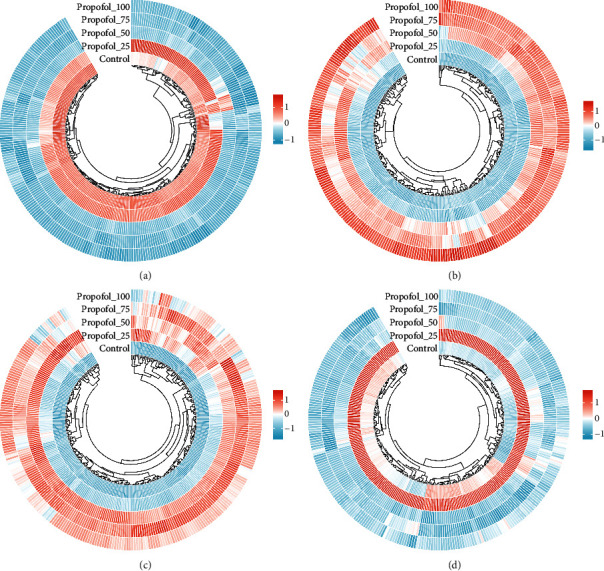
Heat map showing the expression of each gene in each sample. (a) Heatmap of module 0 gene expression in each sample. (b) Heatmap of module 19 gene expression in each sample. (c) Heatmap of module 18 gene expression in each sample. (d) Heatmap of module 12 gene expression in each sample.

Subsequently, we selected statistically significant modules for expression pattern clustering analysis. The heat map shows the expression of each trend module gene in each group of samples (see Figure 3).

### 3.3. WGCNA Analysis

**Figure 4:**
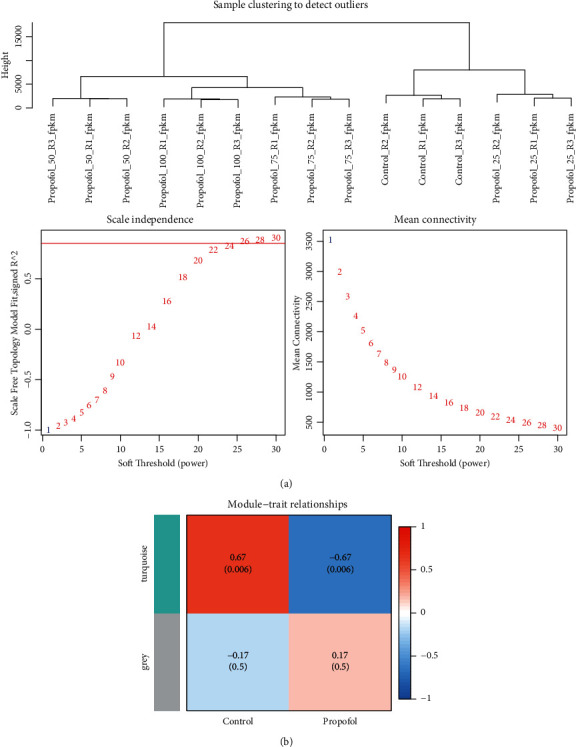
WGCNA analysis. (a) Heat map of correlations between (a) soft threshold (b) module signature genes and propofol treatment groups.

We selected module 0, in which gene expression was downregulated with propofol concentration, and module 19, in which gene expression increased with propofol concentration; we collected genes from both modules for WGCNA analysis. The analysis identified a total of two panels, in which the turquoise panel genes were negatively correlated with the treatment of propofol (see Figure 4).

### 3.4. PPI Network Construction

**Figure 5:**
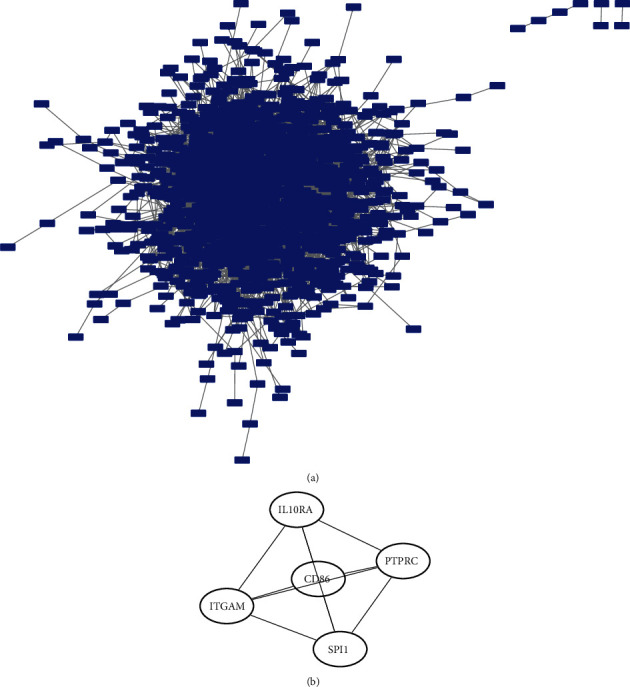
PPI network construction. (a) PPI network building block diagram. (b) 5 key genes obtained from MCC algorithm analysis.

We selected the genes with Cor R >0.9 in the turquoise panel, a total of 1,698 genes, to construct the PPI network. Subsequently, the top 5 key genes, i.e., CD86, IL10RA, PTPRC, SPI1, and ITGAM, were screened using the MCC algorithm (see Figure 5).

### 3.5. GSEA Analysis

**Figure 6:**
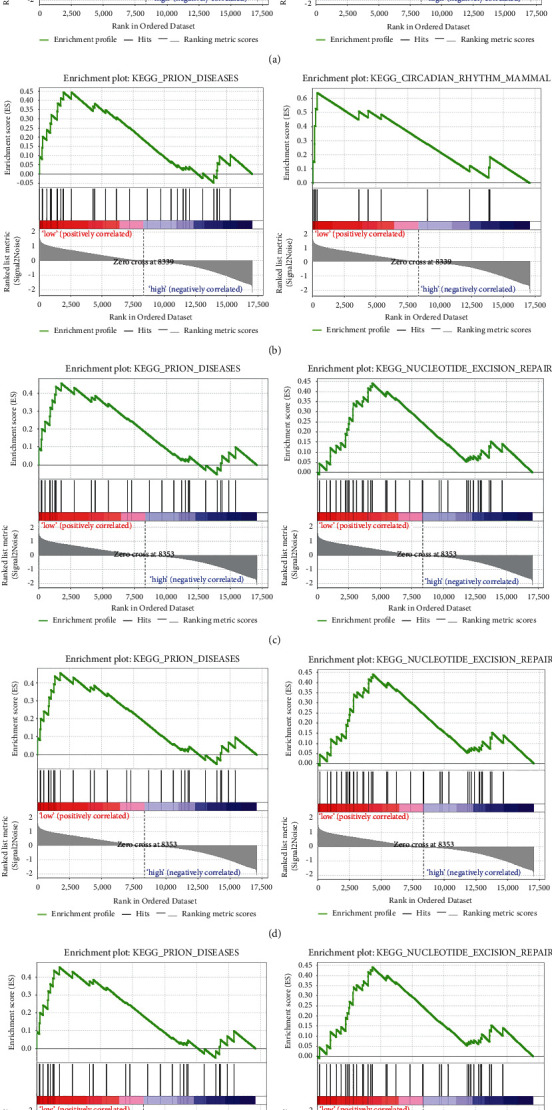
GSEA analysis. (a) The first two signaling pathways involved in CD86 low expression. (b) The first two signaling pathways involved in IL10RA low expression. (c) The first two signaling pathways involved in PTPRC low expression. (d) The first two signaling pathways involved in SPI1 low expression. (e) The first two signaling pathways involved in ITGAM low expression.

We grouped the samples as high and low parts, while GSEA analysis was performed on single genes afterward. The analysis figured out that the low expression of CD86, IL10RA, PTPRC, SPI1, and ITGAM all have important roles in the PRION_DISEASES pathway, and CD86, PTPRC, SPI1, and ITGAM are also all involved in the NUCLEOTIDE_EXCISION_REPAIR signaling pathway. Furthermore, IL10RA is involved in the CIRCADIAN_RHYTHM_MAMMAL signaling pathway (see Figure 6).

## 4. Discussion

Propofol causes fewer anesthetic side effects and facilitates faster recovery compared to other intravenous anesthetics. The benefits of propofol include the rapid onset of anesthesia, short recovery time, and neuroprotective effects in pathogenic situations [8, 10]. Hence, concerns have been raised about the possible effects of the widespread use of propofol on the central nervous system. Propofol does have neurotoxic effects on children. It affects brain development by inhibiting neuronal activation of hippocampal neurons, which may lead to the reduction of neurocognitive function [11]. Animal experiments have shown that exposure to subanesthetic doses of isoproterenol alters the long noncoding RNA profile in the hippocampus of immature mice and leads to disruption of hippocampal circuits, while exposure to high doses of isoproterenol inhibits long-term potentiation in the CA1 region of the adult hippocampus [12]. Previous studies have suggested that this may be because propofol is the most potent drug for activating the GABA-A currents in the immature hypothalamus [13]. To date, the specific molecular mechanisms involved in propofol affecting the hippocampal neuronal cells still remain unclear. To find the potential mechanisms by which propofol may affect hippocampal neuronal cells, we performed a trend analysis of the results obtained from sequencing. We found that 2,506 genes may decrease in expression with increasing propofol concentrations, and 1,871 genes may increase in expression with increasing propofol concentrations. We then subjected these 4,377 genes to WGCNA analysis and found that the turquoise plate genes were significantly and negatively correlated with the propofol in your treatment group. We then selected the genes in this panel with correlation coefficients greater than 0.9 for PPI network construction and screened five key genes: CD86, IL10RA, PTPRC, SPI1, and ITGAM.

CD86 is a type I transmembrane protein associated with T-cell activation in the immune system [14]. CD86 may act as a key regulator of the immune response to disease through a T cell-mediated mechanism, and thus it has great potential to become a new target for immunotherapy [15]. IL10RA consists mainly of a heterotetramer of the anti-inflammatory cytokine IL10 receptor and belongs to class II cytokines [16, 17]. IL10RA is usually located as a cell surface receptor upstream of STAT3, and it can bind IL-10 together with IL10RB to mediate downstream signaling via STAT3 [18]. PTPRC is expressed on all nucleated cells of the hematopoietic system. It expresses several isoforms specific to a certain cell type and cell development or activation state [19]. PTPRC also plays an important role in autoimmune diseases and cancers, as well as in infectious diseases; its deficiency may lead to T and B lymphocyte dysfunction, manifesting as severe combined immunodeficiency [20].

Spi1 is a major regulator of hematopoiesis because it is involved in the self-renewal of hematopoietic stem and progenitor cells, as well as in the stereotyping and maturation of the myeloid and B-lymphatic lineages. Inappropriate Spi1 expression is oncogenic. However, the molecular mechanisms mediating the oncogenic function of Spi1 are complex and not yet fully understood [21]. In addition, Spi1 transcription factors are important in regulating macrophage and neutrophil development [22]. ITGAM is expressed by a variety of myeloid cell types, which can form heterodimers with CD18 and mediate adhesion between cell types in the immune system [23, 24]. ITGAM deficiency may enhance disease progression and inflammation in multiple autoimmune models, including lupus [25]. The above then could show that CD86, IL10RA, PTPRC, SPI1, and ITGAM all play important roles in immune response. Inflammation is a response triggered by damage to living tissue. It can be beneficial or may lead to tissue destruction. Inflammation is particularly dangerous when the nervous system is involved (so-called “neuroinflammation”), whether acute or chronic [26]. In turn, neuroinflammation can cause reversible and irreversible neurological sequelae, including cognitive impairment [27]. However, by modulating inflammation, it is also possible to improve peripheral nerve repair [28]. It has been suggested that the abnormal expression of CD86, IL10RA, PTPRC, SPI1, and ITGAM may trigger an immune-inflammatory response and thus lead to neurological damage.

In the present experiment, we performed GSEA analyses of individual genes separately and showed that CD86, IL10RA, PTPRC, SPI1, and ITGAM are involved in the PRION_DISEASES pathway. Prion diseases can make the prion protein (PrP Sc) in the central nervous system itch. In the early stages, microglia respond to the deposition of PrP Sc, thereby increasing their phagocytic capacity to clear PrP Sc. However, this phagocytosis is not sufficient and can have detrimental effects on the brain [29]. This is further evidence that CD86, IL10RA, PTPRC, SPI1, and ITGAM have an important relationship in neurological impairment of the brain. This could also suggest that propofol may lead to neurological impairment by affecting the abnormal expression of CD86, IL10RA, PTPRC, SPI1, and ITGAM; thus, the exact mechanism remains to be investigated.

## 5. Conclusion

In conclusion, CD86, IL10RA, PTPRC, SPI1, and ITGAM may play an important role in propofol affecting mouse hippocampal neuronal cells. Propofol may affect mouse hippocampal neurons by affecting the expression of PTPRC, CD86, il10ra, spi1il10ra and ITGAM. Thereby, these findings may imply potential new targets for the treatment of patients with propofol anesthesia-induced neurological injury.

## Data Availability

The datasets used and analyzed during the current study are available from the corresponding author on reasonable request.

## References

[1] Sahinovic M. M., Struys M. M. R. F., Absalom A. R. (2018). Clinical pharmacokinetics and pharmacodynamics of propofol. *Clinical Pharmacokinetics*.

[2] Chen L., Yang Z.-l., Cheng J. (2019). Propofol decreases the excitability of cholinergic neurons in mouse basal forebrain via GABAA receptors. *Acta Pharmacologica Sinica*.

[3] Nishizawa T., Suzuki H. (2018). Propofol for gastrointestinal endoscopy. *United European Gastroenterology Journal*.

[4] Xu Y., Pan S., Jiang W., Xue F., Zhu X. (2020). Effects of propofol on the development of cancer in humans. *Cell Proliferation*.

[5] Zhang W., Wang Y., Zhu Z., Zheng Y., Song B. (2018). Retracted: propofol inhibits proliferation, migration and invasion of gastric cancer cells by up-regulating microRNA-195. *International Journal of Biological Macromolecules*.

[6] Mhuircheartaigh R. N., Rosenorn-Lanng D., Wise R., Jbabdi S., Rogers R., Tracey I. (2010). Cortical and subcortical connectivity changes during decreasing levels of consciousness in humans: a functional magnetic resonance imaging study using propofol. *Journal of Neuroscience*.

[7] Zhang S., Liang Z., Sun W., Pei L. (2017). Repeated propofol anesthesia induced downregulation of hippocampal miR-132 and learning and memory impairment of rats. *Brain Research*.

[8] Li X., Yao L., Liang Q., Qu H., Cai H. (2018). Propofol protects hippocampal neurons from hypoxia-reoxygenation injury by decreasing calcineurin-induced calcium overload and activating YAP signaling. *Oxidative Medicine and Cellular Longevity*.

[9] Feng L., Sun Z. G., Liu Q. W. (2020). Propofol inhibits the expression of Abelson nonreceptor tyrosine kinase without affecting learning or memory function in neonatal rats. *Brain and behavior*.

[10] Berndt N., Haq U., Kovács O. (2018). Possible neurotoxicity of the anesthetic propofol: Evidence for the inhibition of complex II of the respiratory chain in area CA3 of rat hippocampal slices. *Archives of Toxicology*.

[11] Yang Y., Yi J., Pan M., Hu B., Duan H. (2021). Edaravone alleviated propofol-induced neurotoxicity in developing Hippocampus by mBDNF/TrkB/PI3K pathway. *Drug Design, Development and Therapy*.

[12] Guan R., Lv J., Xiao F., Tu Y., Xie Y., Li L. (2019). Potential role of the cAMP/PKA/CREB signalling pathway in hypoxic preconditioning and effect on propofolinduced neurotoxicity in the hippocampus of neonatal rats. *Molecular Medicine Reports*.

[13] Tesic V., Joksimovic S. M., Quillinan N. (2020). Neuroactive steroids alphaxalone and CDNC24 are effective hypnotics and potentiators of GABAA currents, but are not neurotoxic to the developing rat brain. *British Journal of Anaesthesia*.

[14] Fang J., Ying H., Mao T. (2017). Upregulation of CD11b and CD86 through LSD1 inhibition promotes myeloid differentiation and suppresses cell proliferation in human monocytic leukemia cells. *Oncotarget*.

[15] Qiu H., Tian W., He Y. (2021). Integrated analysis reveals prognostic value and immune correlates of CD86 expression in lower grade glioma. *Frontiers in Oncology*.

[16] Al-Abbasi F. A., Mohammed K., Sadath S., Banaganapalli B., Nasser K., Shaik N. A. (2018). Computational protein phenotype characterization of IL10RA mutations causative to early onset inflammatory bowel disease (IBD). *Frontiers in Genetics*.

[17] Xue A.-J., Miao S.-J., Sun H. (2020). Intestinal dysbiosis in pediatric Crohn’s disease patients with IL10RA mutations. *World Journal of Gastroenterology*.

[18] Prokoph N., Probst N. A., Lee L. C. (2020). IL10RA modulates crizotinib sensitivity in NPM1-ALK+ anaplastic large cell lymphoma. *Blood*.

[19] Landskron J., Kraggerud S. M., Wik E. (2017). C77G in PTPRC (CD45) is no risk allele for ovarian cancer, but associated with less aggressive disease. *Plos One*.

[20] Rheinländer A., Schraven B., Bommhardt U. (2018). CD45 in human physiology and clinical medicine. *Immunology Letters*.

[21] Rimmelé P., Esposito M., Delestré L. (2017). The Spi1/PU.1 transcription factor accelerates replication fork progression by increasing PP1 phosphatase in leukemia. *Oncotarget*.

[22] Zakrzewska A., Cui C., Stockhammer O. W., Benard E. L., Spaink H. P., Meijer A. H. (2010). Macrophage-specific gene functions in Spi1-directed innate immunity. *Blood*.

[23] Hom G., Graham R. R., Modrek B. (2008). Association of systemic lupus erythematosus withC8orf13-BLKandITGAM-ITGAX. *New England Journal of Medicine*.

[24] Dunne J. L., Collins R. G., Beaudet A. L., Ballantyne C. M., Ley K. (2003). Mac-1, but not LFA-1, uses intercellular adhesion molecule-1 to mediate slow leukocyte rolling in TNF-*α*-induced inflammation. *The Journal of Immunology*.

[25] Ramirez-Bello J., Sun C., Valencia-Pacheco G. (2019). ITGAM is a risk factor to systemic lupus erythematosus and possibly a protection factor to rheumatoid arthritis in patients from Mexico. *Plos One*.

[26] Skaper S. D., Facci L., Zusso M., Giusti P. (2018). An inflammation-centric view of neurological disease: beyond the neuron. *Frontiers in Cellular Neuroscience*.

[27] Alam A., Hana Z., Jin Z., Suen K. C., Ma D. (2018). Surgery, neuroinflammation and cognitive impairment. *EBioMedicine*.

[28] Huang T.-C., Wu H.-L., Chen S.-H., Wang Y.-T., Wu C.-C. (2020). Thrombomodulin facilitates peripheral nerve regeneration through regulating M1/M2 switching. *Journal of Neuroinflammation*.

[29] Aguzzi A., Zhu C. (2017). Microglia in prion diseases. *Journal of Clinical Investigation*.

